# Editorial: Neural mechanisms of language and cognitive performance in individuals with neurodevelopmental disorders

**DOI:** 10.3389/fpsyg.2024.1414354

**Published:** 2024-04-29

**Authors:** Eleni Peristeri, Christos A. Frantzidis, Maria Andreou

**Affiliations:** ^1^Department of English Studies, Aristotle University of Thessaloniki, Thessaloniki, Greece; ^2^UK School of Computer Science, University of Lincoln, Lincoln, United Kingdom; ^3^Department of Speech and Language Therapy, University of Peloponnese, Kalamata, Greece

**Keywords:** autism spectrum disorder, language, cognition, neural mechanisms, neurodevelopmental disorders, reading comprehension

Research has indicated that, as compared to typically developing peers, individuals with autism struggle across multiple domains in both language and cognition. The domains that have been shown to be mostly negatively affected in language is pragmatic language comprehension (e.g., Solari et al., 2019), auditory processing (O'Connor, 2012), and expressive language (Peristeri et al., 2017). Individuals with autism have also been reported to experience difficulties with executive functions (Andreou et al., 2022), intellectual functioning (Peristeri and Andreou, 2024), and social cognition (Andreou et al., 2020; Peristeri et al., 2023), which often impede their daily functioning and adaptive behavior (Tsermentseli et al., 2018). The current Research Topic aims to expand the current understanding of language and cognitive skills of individuals with autism through the utilization of multiple methodological approaches and the consideration of less well-studied language and cognitive phenomena.

Lupi et al.'s study investigates the longitudinal relationships between executive functioning, autism symptomatology, social and personal-emotional adjustment problems in preschool-aged children with autism who were followed up 2 years after initial assessment. Preschoolers with stable difficulties in everyday executive functioning across 2 years exhibited increase in autism severity as compared to their peers with no such difficulties, whose autism severity scores were unaffected over time. Moreover, executive functioning difficulties were associated with the children's social cognition, emotional and behavioral adjustment problems, but not their cognitive development. The study is important in that it examines age-related changes in the executive function skills of young children with autism, and also highlights the way executive functioning transcends social cognition, emotional and behavioral skills that are hallmark difficulties in children with autism.

Demopoulos et al. delve into the relationship between cortical auditory processing and verbal communication abilities in individuals with autism. The research cohort comprised 57 children and adolescents with autism. Utilizing magnetoencephalography, the authors gauged the auditory response quality to rapid speech sound stimuli. Their findings unveiled a connection between impaired rapid auditory processing and deficiencies in the individuals' speech articulation and overall expressive language proficiency. Notably, auditory processing in the left hemisphere was related to expressive language skills and phonological memory, while phonological memory significantly mediated the relationship between cortical processing and receptive language. These insights underscore the effect of auditory processing impairments in the speech production skills of individuals with autism, especially phonological memory and articulatory control.

Irie et al.'s study adopts an embodied approach to the language comprehension skills of young adults with autism by investigating their sensibility judgments for sentences describing actions that matched (or not) the behavior described in the sentences. The action–sentence compatibility effect in their study was realized through the manipulation of adverbs and onomatopoeias in sentences. The authors found that the strength of the action-sentence compatibility effect was critically modulated by the participants' autistic traits. Specifically, the group with low autistic traits was more likely to experience a facilitating effect in their reaction times on the sentence processing task for those trials exhibiting a match between the linguistic description and the physical response associated with word comprehension, while no such effect was observed for the group with high autistic traits. The findings underscore that the coupling of cognition and action may enhance language comprehension in individuals with autism, especially those with low autistic traits.

Cano-Villagrasa et al.'s study delineates the language, cognitive and sensory profile competencies of a child exhibiting symptoms compatible with both autism and epilepsy. Their study reports on considerable language difficulties in the domains of vocabulary and language comprehension, which deteriorated following the child's first epileptic episode. The child also exhibited cognitive rigidity and attentional problems that resulted in disruptive behaviors in novel and unexpected situations, and hyper-sensitivity to touch which was indicative of sensory reactivity disturbances. The findings deepen our understanding of the effects of the co-occurring conditions of autism and epilepsy on the language, cognitive and sensory functioning of individuals, and also underscore the need to design refined evaluation protocols to assess the competencies of individuals with autism and co-occurring medical conditions, including epilepsy.

Peristeri et al.'s work delves into the reading comprehension skills of children with autism and intact or impaired intellectual functioning skills, and it also offers critical insights in the way(s) decoding, fluency, morphological and syntactic, and metalinguistic skills affect reading comprehension in the same children. Findings revealed that children with autism and low cognitive abilities exhibited lower scores in reading comprehension than their peers with intact cognitive skills, however, there was no difference in decoding, verb production, and compound word formation between the two groups. In the group with intact cognitive abilities, reading comprehension was primarily influenced by decoding and fluency skills, whereas for the group with cognitive impairment, reading comprehension performance was significantly affected by decoding, morphosyntax and metalinguistic skills. Notably, reading comprehension was negatively affected across both groups with autism irrespective of cognitive ability, with ~$\frac{1}{2}$ of the children comprising the intact cognitive group encountering mild-to-moderate reading challenges. Decoding boosted reading comprehension for the children with cognitive impairment, whereas fluency and metalinguistic skills were pivotal for those with intact cognition. The study's findings indicate that reading comprehension imposes challenges for children on the spectrum irrespective of their intellectual functioning level, and that decoding, syntax and metalinguistic skills may be necessary for basic skills that support reading comprehension.

The five papers address varied research questions that illuminate the language and cognitive complexities inherent in how individuals with autism navigate their verbal communication and everyday functioning. Combined, the overall findings highlight neurodevelopmental differences at various levels of cognitive functioning of individuals with autism. Importantly, these differences are linked to outcomes across various domains which are important for these individuals' quality of life, and represent potentially suited treatment targets. The dynamic interdependencies between executive functioning, auditory processing and metalinguistic awareness skills, on the one hand, and language and socio-cognitive abilities in individuals with autism, on the other, may be useful for targeting domain-general cognitive, sensory and linguistic disruptions to improve everyday functioning in individuals with autism.

## Author contributions

EP: Writing—original draft, Writing—review & editing. CF: Writing—original draft, Writing—review & editing. MA: Writing—original draft, Writing—review & editing.
